# Characterization of *Staphylococcus aureus* from Humans and a Comparison with İsolates of Animal Origin, in North Dakota, United States

**DOI:** 10.1371/journal.pone.0140497

**Published:** 2015-10-20

**Authors:** Valeria Velasco, Esra Buyukcangaz, Julie S. Sherwood, Ryan M. Stepan, Ryan J. Koslofsky, Catherine M. Logue

**Affiliations:** 1 Department of Veterinary and Microbiological Sciences, North Dakota State University, Fargo, North Dakota, United States of America; 2 Department of Animal Sciences, University of Concepción, Chillán, Chile; 3 Department of Microbiology, College of Veterinary Medicine, Uludag University, Bursa, Turkey; 4 Department of Veterinary Microbiology and Preventive Medicine, College of Veterinary Medicine, Iowa State University, Ames, Iowa, United States of America; University of Iowa Carver College of Medicine, UNITED STATES

## Abstract

Different clones of methicillin-susceptible (MSSA) and methicillin-resistant (MRSA) *Staphylococcus aureus* have been found in humans as well as in animals and retail meat. However, more information about the genetic characteristics and similarities between strains is needed. The aim of this study was to identify and characterize *Staphylococcus aureus* from humans, and to compare their characteristics with isolates of animal origin. A total of 550 nasal swabs were taken from healthy humans, and *S*. *aureus* was isolated and identified. Positive *S*. *aureus* isolates were subjected to molecular typing and susceptibility testing. In addition, 108 MRSA isolates recovered from clinical patients in the state of North Dakota and 133 *S*. *aureus* isolates from animals and meat previously analyzed were included. The nasal carriage of *S*. *aureus* in healthy people was 7.6% and, in general, clones were genetically diverse. None of the *S*. *aureus* strains obtained from healthy people were *mecA*- or PVL-positive. A total of 105 (97.2%) MRSA isolates from clinical cases harbored the *mecA* gene and 11 (10.2%) isolated from blood stream infections harbored the PVL gene. The most common resistance profile among *S*. *aureus* from healthy people was penicillin, and from clinical cases were erythromycin-penicillin-ciprofloxacin. The rate of multidrug resistance (MDR) was 70% in humans. Most of the *S*. *aureus* harboring *mecA* and PVL genes were identified as ST5 and ST8, and exhibited MDR. However, *S*. *aureus* isolates of animal origin used for comparison exhibited a lower rate of MDR. The most common resistance profiles in isolates of animal origin were penicillin-tetracycline and penicillin-tetracycline-erythromycin, in animals and raw meat, respectively. The ST5 was also found in animals and meat, with ST9 and ST398 being the major clones. The genetic similarity between clones from humans and meat suggests the risk of spread of *S*. *aureus* in the food chain.

## Introduction

In the last few decades, many bacterial species have developed resistance to antimicrobial agents that have been commonly used to treat them [1]. *Staphylococcus aureus* is one of the pathogens known to rapidly develop resistance to antimicrobial agents as new antibiotics are introduced [2]. Within a couple years after the introduction of penicillin to clinical medicine, the first penicillin-resistant *S*. *aureus* was discovered. The first methicillin-resistant *S*. *aureus* (MRSA) strains were identified from clinical specimens in 1961; two years after methicillin was introduced as an antibiotic [3, 4].

Methicillin-resistant *S*. *aureus* has been implicated in community-associated (CA-MRSA), healthcare-associated (HA-MRSA), and livestock-associated (LA-MRSA) infections worldwide. In the United States, the nasal carriage of *S*. *aureus* in humans was 29% (78.9 million people) and that of MRSA approximately 1.5% (4.1 million people) in 2003–2004 [5]. In 2005, there were an estimated 478,000 hospitalizations that corresponded to *S*. *aureus* infections, approximately 278,000 of those were attributed to MRSA [6]. In addition, an invasive MRSA infection was developed by about 94,000 people, leading to 19,000 deaths. The distribution of these infections were approximately 86% HA-MRSA and 14% CA-MRSA [7]. However, HA-MRSA clones have been progressively replaced by CA-MRSA strains due to the expanding community reservoir and the increasing influx into the hospital of individuals who harbor CA-MRSA [8, 9].

Meat-producing animals have also been identified as carriers of MRSA [10–12]. Moreover, it has been found that retail meat can also be contaminated with MRSA [13–15]. These findings have increased the concern that food may serve as a vehicle for transmission of MRSA to the human population [16].

Resistance to methicillin in *S*. *aureus* is primarily mediated by the *mecA* gene, which encodes the low-affinity penicillin-binding protein 2a (PBP2a) [17, 18]. Recently, a novel *mecA* homolog gene (*mecA*_LGA251_ re-named *mecC*) has been detected in *S*. *aureus* strains from humans and livestock that were phenotypically resistant to methicillin but tested negative for the *mecA* gene. The *mecC* gene exhibits about 70% sequence homology to the *mecA* gene and is located on the staphylococcal cassette chromosome *mec* type XI (type-XI SCC*mec*) [19–22]. Among virulence factors, the Panton–Valentine leukocidin (PVL) exotoxin encoding gene has been associated with most CA-MRSA strains [23, 24] that cause severe skin infections and necrotizing pneumonia [25].

Different clones of methicillin-susceptible *S*. *aureus* (MSSA) and MRSA have been found in humans as well as in animals and retail meat. Typically, clones that cause CA-MRSA infections (USA300 and USA400) are different than those causing HA-MRSA infections (USA100 and USA200) [26]. Some sequence types (ST) associated to HA-MRSA have been identified including: ST5, ST8, ST22, ST36, ST45, among others [27]. The sequence types ST30 and ST80 have been associated with CA-MRSA [28] and ST398 has been linked with animals [10, 29]. The sequence types ST398 and ST9 have been detected in both animals (pigs) and meat (pork meat), with a genetic similarity between *S*. *aureus* strains from these different sources [15]. However, the clonal type ST398 has also been detected in human patients [10, 29].

The objective of this study was to identify and characterize *Staphylococcus aureus* isolated from humans, and to compare the molecular characteristics and antimicrobial susceptibility with *S*. *aureus* isolates from animals and meat.

## Materials and Methods

### Samples

A total of 550 nasal swab samples were obtained from undergraduate students enrolled in the Department of Veterinary and Microbiological Sciences, North Dakota State University, who were considered as healthy humans. Samples were obtained from plates used in class that were discarded at the end of the study, thus none of the isolates obtained were identifiable by traceback. Samples were collected in the fall semester of 2010 (*n* = 231) and in the spring semester of 2011 (*n* = 319). In addition, a total of 108 MRSA isolates recovered from clinical cases of MRSA affected by wound and blood stream infections (sepsis, bone, cerobrospinal fluid [CSF], synovial fluid, subdural fluid, tissue, leg ulcer and pleural fluid) were obtained from the North Dakota Department of Health (Bismarck, ND) in the summer of 2010.

A total of 133 *S*. *aureus* strains isolated from animals (pig, *n* = 30; sheep, *n* = 26; cattle, *n* = 2), raw meat (pork, *n* = 35; chicken, *n* = 25; beef, *n* = 9), and deli meat (ham, *n* = 4; chicken, *n* = 2) were used to compare the molecular characteristics and antimicrobial susceptibility with *S*. *aureus* isolates from humans. The *S*. *aureus* strains of animal origin were isolated and analyzed as previously described by Buyukcangaz *et al*. (2013) [15] (Tables 1 and 2).

**Table 1:** Source and characteristics of *S*. *aureus* isolates of animal origin used in the study.

Source	*S*. *aureus* isolates	16S rRNA	*mecA*	PVL
**Animals**	----------No.----------	------------------------No.------------------------		
** Sheep**	26	26	0	0
** Pig**	30	30	0	0
** Cattle**	2	2	0	0
**Total**	58	58	0	0
**Raw meat**				
** Pork**	35	35	5	0
** Chicken**	25	25	0	0
** Beef**	9	9	0	0
**Total**	69	69	5	0
**Deli meat**				
** Ham**	4	4	0	0
**Turkey**	0	0	0	0
**Chicken**	2	2	0	0
**Total**	6	6	0	0

Adapted from Buyukcangaz *et al*. (2013) [15].

**Table 2:** Antimicrobial resistance profiles of *S*. *aureus* isolates of animal origin used in this study.

Antimicrobial resistance profile	Antimicrobial subclasses	Isolates	Source	Isolates
	----------No.----------	--No.--	-----------------No.-----------------	
**ERY-PEN-TET-GEN-CHL-CIP-QUI**	7	1	Pork meat	1
**ERY-PEN-TET-CHL-CIP-QUI**	6	1	Pork meat	1
**ERY-PEN-TET-CHL**	4	2	Pig	2
**ERY-PEN-TET-KAN**	4	1	Pork meat	1
**ERY-PEN-TET**	3	14	Pork meat	11
				3^a^
**PEN-TET-GEN**	3	1	Sheep	1
**PEN-TET-KAN**	3	1	Pork meat	1^a^
**PEN-TET-CHL**	3	1	Pig	1
**ERY-PEN**	2	4	Pork meat	3
				1^a^
**ERY-TET**	2	5	Pork meat	5
**PEN-TET**	2	39	Pig	19
			Sheep	17
			Pork meat	2
			Chicken meat	1
**ERY**	1	4	Chicken meat	3
			Chicken deli meat	1
**PEN**	1	21	Pig	7
			Sheep	1
			Pork meat	4
			Beef	4
			Chicken meat	3
			Ham	2
**TET**	1	10	Sheep	6
			Pork meat	3
			Chicken meat	1

Ciprofloxacin (CIP); chloramphenicol (CHL); erythromycin (ERY); gentamicin (GEN); kanamycin (KAN); quinupristin/dalfopristin (QUI); penicillin (PEN); and tetracycline (TET).

^a^ *mecA* positive.

Adapted from Buyukcangaz *et al*. (2013) [15], considering the resistance according to CLSI (2012) criteria [34].

Institutional Review Board (IRB) approval was sought for the human isolates and the study was considered exempt by NDSU IRB. Institutional Animal Care and Use Committee (IACUC) approval was used for the animal work as described previously [15].

### Culture method

Nasal swabs were taken from healthy humans by using a sterile moistened swab inserted into the nostril, to a depth of approximately 1 cm, and rotated five times. For each subject, both nostrils were sampled using the same swab. Nasal swabs were inoculated onto mannitol salt agar (MSA) plates (Becton, Dickinson and Company [BD], Sparks, MD) and incubated at 37°C for 48 h. All colonies surrounded by yellow zones on MSA after incubation were selected. Colonies with pink or red zones on MSA were excluded.

All presumptive *S*. *aureus* colonies were confirmed by biochemical testing using Sensititre Gram Positive ID (GPID) plates (Sensititre^®^, TREK Diagnostic Systems Ltd., Cleveland, OH), according to the manufacturer's recommendations.

*Staphylococcus aureus* isolates from healthy humans, and MRSA isolates from clinical cases, were stored at -80°C in brain–heart infusion broth (BD) containing 20% glycerol until use.

### Multiplex polymerase chain reaction (mPCR)

*Staphylococcus aureus* strains from healthy humans and from clinical cases stored at -80°C were recovered to trypticase soy agar (TSA) plates and incubated at 37°C for 18 to 24 h. The extraction of DNA was carried out by suspending one colony in 50 μL of DNase/RNase-free distilled water (Gibco Invitrogen, Grand Island, NY, USA), heating (99°C, 10 min) and centrifugation (30,000 × *g*, 1 min) to remove cellular debris. The remaining DNA was transferred to a new tube and stored at -20°C until use.

A multiplex PCR assay was used to detect: 16S rRNA (identification of *S*. *aureus*), *mecA* (associated with methicillin resistance) and PVL-encoding genes (virulence factor) (Table 3). Two microliters of the DNA template (described above) was added to a 50 μL final reaction mixture: 1X Go Taq^®^ Reaction Buffer (pH 8.5), 1.25 U of Go Taq^®^ DNA polymerase, 200 μM dNTP (Promega, Madison, WI, USA), and 1 μM of primers (16S rRNA, *mecA*, LukS/F-PV) (Integrated DNA Technologies, Inc., Coralville, IA, USA). The conditions of the PCR reactions were adjusted according to the protocol described by Makgotlho *et al*. (2009) [30] using a thermocycler (Eppendorf, Hamburg, Germany).

**Table 3:** Nucleotide sequence of the primers used for detection of 16S rRNA, *mecA*, Panton-Valentine leukocidin, *mecA*_LGA251_, *arcC*, *aroE*, *glpF*, *gmk*, *pta*, *tpi*, and *yqiL* genes.

Primer	Oligonucleotide sequence	Amplicon Size (bp)
**Staph 756 F**	5’-AACTCTGTTATTAGGGAAGAACA-3’	756
**Staph 750 R**	5’-CCACCTTCCTCCGGTTTGTCACC-3’	
***mecA* 1 F**	5’-GTAGAAATGACTGAACGTCCGATAA-3’	310
***mecA*-2 R**	5’-CCAATTCCACATTGTTTCGGTCTAA-3’	
***luk*-PV-1 F**	5’-ATCATTAGGTAAAATGTCTGGACATGATCCA-3’	433
***luk*-PV-2 R**	5’-GCATCAAGTGTATTGGATAGCAAAAGC-3’	
***mecA***_**LGA251**_ **FP**	5'-TCACCAGGTTCAAC[Y]CAAAA-3'	356
***mecA***_**LGA251**_ **RP**	5'-CCTGAATC[W]GCTAATAATATTTC-3'	
***mecA***_**LGA251**_ **MultiFP**	5'-GAAAAAAAGGCTTAGAACGCCTC-3'	718
***mecA***_**LGA251**_ **RP**	5'-CCTGAATC[W]GCTAATAATATTTC-3'	
***mecA***_**LGA251**_ **MultiFP**	5'-GAAAAAAAGGCTTAGAACGAATC-3'	138
***mecA***_**LGA251**_ **MultiRP**	5'-GATCTTTTCCGTTTTCAGC-3'	
***arcC F***	5'-TTGATTCACCAGCGCGTATTGTC-3'	456
***arcC R***	5'-AGGTATCTGCTTCAATCAGCG-3'	
***aroE F***	5'-ATCGGAAATCCTATTTCACATTC-3'	456
***aroE R***	5'-GGTGTTGTATTAATAACGATATC-3'	
***glpF F***	5'-CTAGGAACTGCAATCTTAATCC-3'	465
***glpF R***	5'-TGGTAAAATCGCATGTCCAATTC-3'	
***gmK F***	5'-ATCGTTTTATCGGGACCATC-3'	429
***gmK R***	5'-TCATTAACTACAACGTAATCGTA-3'	
***pta F***	5'-GTTAAAATCGTATTACCTGAAGG-3'	474
***pta R***	5'-GACCCTTTTGTTGAAAAGCTTAA-3'	
***tpi F***	5'-TCGTTCATTCTGAACGTCGTGAA3'	402
***tpi R***	5'-TTTGCACCTTCTAACAATTGTAC-3'	
***yqiL F***	5'-CAGCATACAGGACACCTATTGGC-3'	516
***yqiL R***	5'-CGTTGAGGAATCGATACTGGAAC-3'	

16S rRNA, *mecA*, and Panton-Valentine leukocidin genes [47].

*mecA*_LGA251_ gene [31].

*arcC*, *aroE*, *glpF*, *gmk*, *pta*, *tpi*, and *yqiL* genes [33].

The mPCR products (10 μL) were loaded into a 1.5% (wt/vol) agarose gel (Agarose I^TM^) using EzVision One loading dye (Amresco, Solon, OH, USA) and electrophoresis was carried out in 1X TAE buffer at 100 v for 1 h. A molecular weight marker 100-bp ladder (Promega, Madison, WI, USA) and a negative (DNase/RNase-free distilled water) and a positive control (*S*. *aureus* ATCC 33591; MRSA) were included on each gel. Bands corresponding to each gene were visualized using an Alpha Innotech UV imager (FluorChem^TM^).

All MRSA clinical isolates that were negative for the *mecA* gene by mPCR assay were subjected to the detection of the *mecC* gene (Table 3) by PCR according to the protocol described by Stegger *et al*. (2011) [31].

### Pulsed-field gel electrophoresis (PFGE)

The PulseNet protocol with minor modifications was used [26]. Briefly, *S*. *aureus* strains were recovered from frozen stock to TSA plates and incubated at 37°C for 18 to 24 h. A single colony was inoculated onto a second TSA plate and incubated at 37°C for 18 to 24 h. Colonies were transferred to 5-mL polystyrene round-bottom tubes containing 2 mL of cell suspension buffer (100 mM Tris HCl [pH 8.0], Invitrogen; 100 mM EDTA [pH 8.0], Gibco), adjusting the cell concentrations to an absorbance of 0.9 to 1.1 using a spectrophotometer (Smart SpecTM plus, Bio-Rad Laboratories, USA) at 610 nm. The following steps (plug preparation, lysis, washing, and the *SmaI* enzyme restriction digestion) were performed according to the PulseNet protocol. *Salmonella* Branderup H9812 was used as a DNA marker [32].

The electrophoresis was carried out in a Chef Mapper (Bio-Rad Laboratories) PFGE rig, with an initial switch time of 5 s, a final switch time of 40 s, and a total running time of 17 h 45 min. The gels were stained with ethidium bromide (1.5 μg/mL), and then the macrorestriction patterns were visualized using a UVP imager (UVP, Upland, CA).

Macrorestriction patterns of *Staphylococcus aureus* isolates from humans, animals and meat were analyzed using the BioNumerics Fingerprinting software (Ver 6.6 Applied Math, Austin, TX). The similarity index was calculated using the Dice coefficient, a band position tolerance of 1%, and an optimization of 0.5%. The unweighted-pair group method with arithmetic mean algorithm (UPGMA) was used to construct a dendrogram, and clusters were selected using a cutoff at 80% level of genetic similarity [26].

### Multilocus sequence typing (MLST)

After the construction of the dendrogram (PFGE) containing *S*. *aureus*, at least one human isolate from each cluster was selected as a representative of the group for MLST analysis. Strains of *S*. *aureus* from animals and meat were included for comparison and STs were obtained from previous work [15]. Sequencing of MLST PCR products of the selected human isolates was carried out at Iowa State University’s DNA Sequencing Facility (Ames, IA). Briefly, *S*. *aureus* isolates were struck to TSA plates and incubated at 37°C for 18 to 24 h. DNA extraction from cells was carried out using the boiling method as described above.

Internal fragments of the following seven housekeeping genes: *arcC*, *aroE*, *glpF*, *gmk*, *pta*, *tpi*, and *yqiL* were amplified (Table 3; S1 Data) [33]. All PCR reactions were carried out in 50-μL volumes: 1 μL of DNA template, Taq DNA polymerase (Promega) (1.25 U), 1X PCR buffer (Promega), primers (0.1 μM) (Integrated DNA Technologies, Inc.), and dNTPs (200 μM) (Promega). The PCR conditions were adjusted according to the protocol described by Enright *et al*. (2000) [33] using a thermocycler (Eppendorf). Ten microliters of the PCR products were loaded into 1% agarose gels in 1X TAE with EzVision One loading dye, and electrophoresis was run at 100 V in 1X TAE for 1 h. Images were captured using an Alpha Innotech imager.

The amplicon purification was carried out using the QIAquick^®^ PCR Purification Kit (Qiagen, Valencia, CA) according to the manufacturer’s instructions. Purified PCR products were sequenced at Iowa State University’s DNA Facility (Ames, IA) using an Applied Biosystems 3730xl DNA Analyzer (Applied Biosystems, Foster City, CA). Sequence data were imported into DNAStar (Lasergene, Madison, WI), trimmed, and aligned to the control sequences (from the MLST site) and interrogated against the MLST database (http://saureus.mlst.net/). Sequence types of selected *S*. *aureus* isolates were added to the strain information for analysis in BioNumerics software.

### Susceptibility testing

*Staphylococcus aureus* isolates were subjected to antimicrobial susceptibility testing using the broth microdilution method and the National Antimicrobial Resistance Monitoring System (NARMS) panels (CMV3AGPF, Sensititre^®^, Trek Diagnostics), according to the manufacturer’s and the Clinical Laboratory Standards Institute (2012) [34] guidelines. Antimicrobials in the panel and their resistance breakpoints were as follows: erythromycin (≥8 μg/mL), tetracycline (≥16 μg/mL), ciprofloxacin (≥4 μg/mL), chloramphenicol (≥32 μg/mL), penicillin (≥0.25 μg/mL), vancomycin (≥16 μg/mL), nitrofurantoin (≥128 μg/mL), gentamicin (≥16 μg/mL), quinupristin/dalfopristin (≥4 μg/mL), linezolid (≥8 μg/mL), kanamycin (≥64 μg/mL), and daptomycin (susceptible ≤1 μg/mL). Multidrug resistance (MDR) was considered as resistance to at least three classes of the antimicrobials tested [35] (S4 Data).

## Results

The results for identification of *S*. *aureus* (16S rRNA), *mecA* and PVL genes in samples obtained from humans are shown in Table 4 and S2 Data & S3 Data. The prevalence of nasal carriage of *S*. *aureus* in healthy people was 7.6%. None of these isolates harbored the *mecA* or PVL genes. Clinical isolates were identified as MRSA strains in the hospital using standard microbiological procedures. As expected, all of these isolates were confirmed as *S*. *aureus* strains by the detection of 16S rRNA gene using the PCR assay. Among the 108 MRSA clinical isolates, a total of 105 (97.2%) harbored the *mecA* gene and 11 (10.2%) carried the PVL gene. Of interest, the PCR assay did not detect the PVL gene in MRSA strains isolated from clinical cases affected by wound infections.

**Table 4:** Identification of 16S rRNA, *mecA* and Panton-Valentine Leukocidin (PVL) genes in *S*. *aureus* from healthy people, and MRSA isolates from clinical cases.

Source	Samples	Positive for *S*. *aureus*	Positive for MRSA	16S rRNA	*mecA*	PVL
**Healthy people**	---No.---	-----No.-----	--No. (%)--	----------------No. (%)----------------		
** Fall 2010**	231	17 (7.4)		17 (7.4)	0 (0.0)	0 (0.0)
** Spring 2011**	319	25 (7.8)		25 (7.8)	0 (0.0)	0 (0.0)
**Total**	550	42 (7.6)		42 (7.6)	0 (0.0)	0 (0.0)
**Clinical cases**						
** Blood**	99	99 (100)	99 (100)	99 (100)	96 (97.0)	11 (11.1)
** Wound**	9	9 (100)	9 (100)	9 (100)	9 (100)	0 (0.0)
**Total**	108	108 (100)	108 (100)	108 (100)	105 (97.2)	11 (10.2)

**Fig 1:**
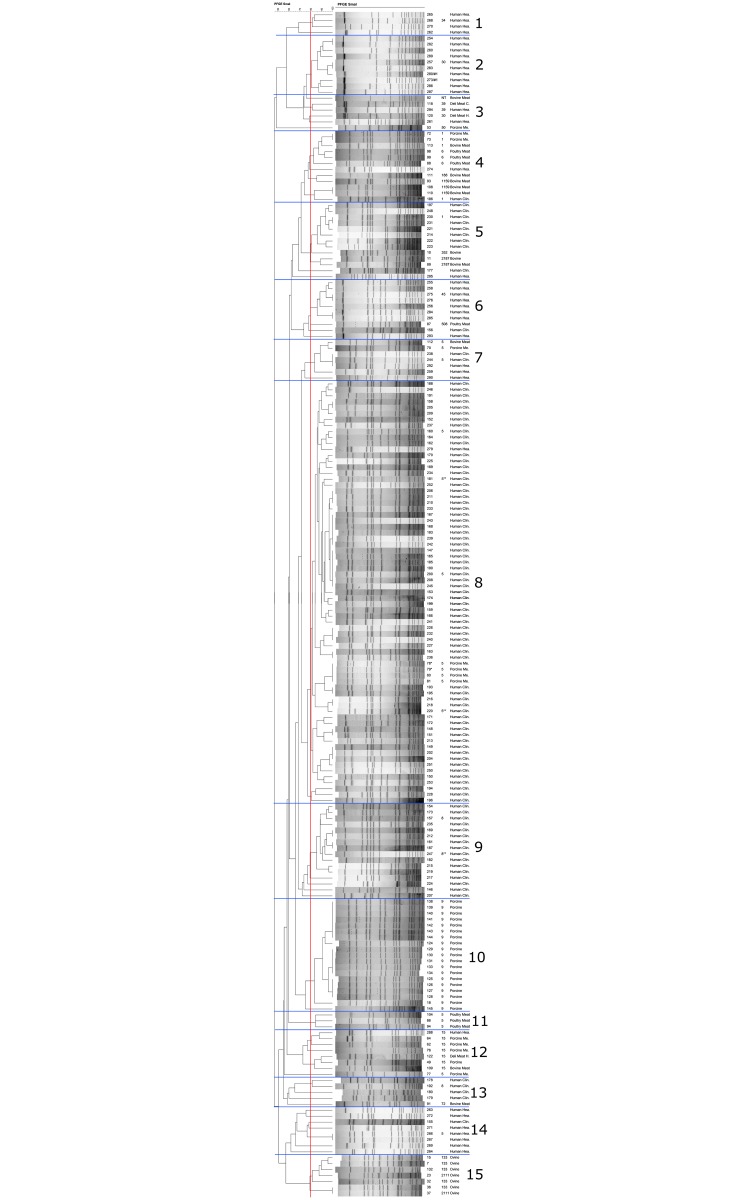
Dendrogram showing the genetic similarity between *S*. *aureus* isolates from humans, and of animal origin. The scale indicates levels of similarity with a vertical line indicating the cutoff (80% level of similarity), numbers represent the sample codes, followed on the right by the sequence type (ST) and the type of the sample. *mecA-positive *S*. *aureus* in pork meat. **mecA- and *mecC*-negative MRSA from clinical cases.

The genetic similarity between *S*. *aureus* strains isolated from humans and *S*. *aureus* strains of animal origin were analyzed using BioNumerics software. Fig 1 shows a dendrogram containing the macrorestriction patterns of *S*. *aureus* strains and the sequence type (ST) of some isolates from each cluster (S1 Data). Thirty-four *S*. *aureus* ST398 strains of animal origin were not included in the dendrogram as they failed to restrict. A total of fifteen clusters was observed, of which six were homogenous, containing one type of isolate exclusively from healthy humans (cluster 1 and 2), MRSA isolates from clinical cases (cluster 9), or isolates of animal origin (clusters 10, 11 and 15). In general, genetic diversity was observed among isolates from healthy humans, classified in different clusters with the sequence types: ST5, ST15, ST30, ST34, ST39 and ST45. Genetic similarity was observed between *S*. *aureus* strains from humans and meat: cluster 3 (ST39), cluster 4 (ST1), cluster 7 (ST5), and cluster 12 (ST15). In cluster 9, the genetic similarity between *mecA*-positive strains and one strain that did not harbor *mecA* nor *mecC* genes isolated from clinical cases, and were identified as ST8. In addition, two clinical isolates identified as MRSA ST5 that were *mecA*- and *mecC*-negative exhibited a genetic similarity with *mecA*-positive *S*. *aureus* ST5 strains isolated from humans and from pork meat (cluster 8).

Among the antimicrobials tested using CLSI interpretation criteria [34], most MRSA isolates from clinical cases were resistant to erythromycin, penicillin and ciprofloxacin, and *S*. *aureus* isolates from healthy people exhibited resistance primarily to penicillin (Table 5, S4 Data). A rate of 70% of MDR strains was detected in humans, primarily among clinical isolates that were all identified as MRSA. In humans, one clinical isolate identified as MRSA was susceptible to all antimicrobial agents. The minimum inhibitory concentrations (MICs) of resistant *S*. *aureus* strains from humans are shown in Table 6. High MICs were observed in most of the penicillin-resistant *S*. *aureus* isolates from humans (8 - >16 μg/mL). The majority of ciprofloxacin-resistant *S*. *aureus* isolates from humans exhibited a MIC > 4 μg/mL.

**Table 5:** Antimicrobial resistance profiles of *Staphylococcus aureus* from healthy people, and methicillin-resistance *Staphylococcus aureus* (MRSA) isolates from clinical cases.

Antimicrobial resistance profile	Antimicrobial subclasses	Samples	Type	Samples
	-----------No.----------	--No.--	---------------No.---------------	
**ERY-PEN-TET-CIP-KAN**	5	5	Clinical MRSA^a^	2
			Healthy human	3
**ERY-PEN-CIP-KAN-QUI**	5	1	Clinical MRSA^a^	1
**ERY-PEN-CIP-KAN-DAP**	5	5	Clinical MRSA^a^	5
**ERY-PEN-CIP-KAN**	4	29	Clinical MRSA^a^	25
			Clinical MRSA^b^	2
			Healthy human	2
**ERY-PEN-CIP-DAP**	4	1	Clinical MRSA^a^	1
**ERY-PEN-TET**	3	2	Healthy human	2
**ERY-PEN-KAN**	3	3	Clinical MRSA^a^	2
			Healthy human	1
**ERY-CIP-KAN**	3	1	Clinical MRSA^a^	1
**PEN-TET-CIP**	3	1	Clinical MRSA^a^	1
**PEN-CIP-KAN**	3	2	Clinical MRSA^a^	1
			Healthy human	1
**ERY-PEN-CIP**	3	55	Clinical MRSA^a^	51
			Healthy human	4
**ERY-PEN**	2	9	Clinical MRSA^a^	36
			Healthy human	6
**PEN-CIP**	2	8	Clinical MRSA^a^	3
			Healthy human	5
**ERY-CIP**	2	1	Clinical MRSA^a^	1
**ERY**	1	2	Healthy human	2
**PEN**	1	22	Clinical MRSA^a^	6
			Healthy human	16
**CIP**	1	2	Clinical MRSA^a^	2

Ciprofloxacin (CIP); Daptomycin (DAP); erythromycin (ERY); kanamycin (KAN); quinupristin/dalfopristin (QUI); penicillin (PEN); and tetracycline (TET).

^a^ *mecA* positive.

^b^ *mecA* and *mecC* negative.

Resistance according to CLSI (2012) criteria [34].

**Table 6:** Minimum inhibitory concentrations (MICs) of resistant *Staphylococcus aureus* isolates from healthy humans and clinical cases.

Antimicrobial Agent (breakpoints)^a^	Resistant *S*. *aureus* isolates	MIC (μg/mL)													
		0.5–1	2	4	>4	8	>8	16	>16	32	>32	256	512	1024	>1024
	----No.----							No.							
								(%)							
**ERY**						7	107								
**(≥8 μg/mL)**						(6.1)	(93.9)								
**PEN**	143	11	9	26		36		36	25						
**(≥0.25 μg/mL)**		(7.7)	(6.3)	(18.2)		(25.2)		(25.2)	(17.5)						
**TET**	8							2		2	4				
**(≥16 μg/mL)**								(25.0)		(25.0)	(50.0)				
**KAN**	46											13	10	13	10
**(≥64 μg/mL)**												(28.3)	(21.7)	(28.3)	(21.7)
**CIP**	111			1	110										
**(≥4 μg/mL)**				(0.9)	(99.1)										
**QUI**	1								1 (100)						
**(≥4 μg/mL)**															
**DAP** ^**b**^	6		3	3											
			(50)	(50)											

Ciprofloxacin (CIP); Daptomycin (DAP); erythromycin (ERY); kanamycin (KAN); quinupristin/dalfopristin (QUI); penicillin (PEN); and tetracycline (TET).

^a^Levels of MIC against tested antibiotics [34].

^b^Criteria for Dap: susceptible ≤1 μg/mL.

## Discussion

Presumptive *S*. *aureus* isolates on MSA plates from healthy people were confirmed by biochemical testing (Sensititre identification plates) with an agreement of 100% with PCR (detection of the 16S rRNA gene) (Table 4). In this study, a nasal carriage rate of 7.6% was observed for *S*. *aureus*, which is considerably lower than the prevalence reported in other studies (29–32%) [5, 36]. However, those studies considered a significantly larger sample size, different demographic characteristics, and different sampling years as part of a nationally representative assessment of carriage of *S*. *aureus*. In this study, *S*. *aureus* strains isolated were from healthy college age individuals and did not harbor the *mecA* or PVL genes. Other studies have reported a nasal carriage rate of MRSA of approximately 0.8 to 1.5% in the community [5, 36], 0.5 to 44% in patients [37], 20% in healthcare workers [38] and 30% in people living and working on farms with MRSA-positive pigs or dust [39]. In contrast, Buyukcangaz *et al*. (2013) [15] failed to detect the *mecA* and PVL genes in *S*. *aureus* isolates from meat-producing animals (Table 1) that were used for the comparison with human isolates in this study. However, a low prevalence of *S*. *aureus* harboring the *mecA* gene was found in pork meat.

The proportion of MRSA in relation to all *S*. *aureus* strains causing infections is still unknown, making it difficult to accurately estimate the magnitude of MRSA infections and to design appropriate health action policies [7, 40]. In this study, three clinical isolates identified as MRSA were negative for the *mecA* gene using the protocol described by Makgotlho et al. (2009) [30]. For that reason, the presence of the novel *mecA* homolog (*mecA*_LGA251_ or *mecC*), was assessed using the protocol described by Stegger et al. (2011) [31]. However, these strains were negative for the *mecC* gene (138 and 718 bp fragments), but tested positive for the 356 bp fragment using degenerate primers. Therefore, further investigation should be carried out to determine the genetic variation of this *mecA* variant. In addition, it is known that borderline oxacillin-resistant *S*. *aureus* (BORSA) exhibit an intermediate resistance level to oxacillin, which is non-*mecA* mediated [41, 42]. All *mecA*- and *mecC*-negative *S*. *aureus* strains identified as MRSA were subjected to oxacillin susceptibility testing. One of those isolates exhibited an intermediate resistance level to oxacillin (2–4 μg/mL) [34], which could be considered as BORSA. Different modifications in the PBP genes causing amino acid substitutions in the transpeptidase domain has been also associated with the borderline resistance [41].

The virulence factor PVL, was detected in this study in 11.1% of MRSA isolates from clinical cases identified as blood stream infections. The MRSA isolates from cases identified as wound infections did not harbor the PVL gene. The PVL toxin is a pore-forming protein that appears to be associated with increased disease severity of *mecA*-positive *S*. *aureus* strains, primarily associated with blood stream infections [24]. Although the PVL gene is considered as a stable marker for CA-MRSA, some CA-MRSA strains have been found to be PVL-negative [9].

In this study, some *S*. *aureus* strains isolated from humans of each cluster in the dendrogram (Fig 1) were subjected to MLST to determine the sequence type. In general, different clones were observed in healthy humans, which indicate the presence of genotypically diverse *S*. *aureus* clones circulating in the community. Although, MRSA was not detected in healthy people, they have the potential to become carriers with the risk of spreading infections to the community [38]. Methicillin-resistant *S*. *aureus* strains isolated from clinical cases in this study presented a lower genetic diversity, and were primarily of ST5 and ST8. Previously, both ST5 and ST8 have been associated with HA-MRSA infections [27, 42]. A description of the genetic characteristics of MRSA clones associated with invasive human infections could help to focus efforts towards the study of the most common clones circulating in the hospital environment.

The molecular characteristics of *S*. *aureus* strains isolated from humans were also compared with isolates of animal origin. Genetic similarity was observed between *mecA*- and *mecC*-negative MRSA isolates from clinical cases and *mecA*-positive *S*. *aureus* strains isolated from clinical cases and pork meat, which suggests modifications occurring in the PBP genes in *mecA*- and *mecC*-negative MRSA strains [41] potentially resulting in slight changes in the macrorestriction patterns observed.

Contamination of meat with *S*. *aureus* strains from animals and humans could occur during slaughtering or processing. In this study, the genetic similarity between strains from humans and meat suggests contamination of raw meat during handling. In addition, the genetic similarity between *S*. *aureus* strains isolated from meat-producing animals and retail meat has been found previously, also supporting contamination of meat during slaughtering [15]. In this study, other *S*. *aureus* strains that have been previously related to LA-MRSA and pig farmers, such as ST398 and ST9 [10, 15, 29] were not detected in *S*. *aureus* isolates from humans. However, Sung *et al*. (2008) [43] found that animal lineages were closely related to human lineages, which could be due to the adaptive behavior of *S*. *aureus* [44].

In this study, resistance to penicillin predominated in the *S*. *aureus* strains isolated from healthy people, and the resistance profiles Ery-Pen-Cip and Ery-Pen-Cip-Kan were most common in MRSA isolates from clinical cases (Table 5). Comparing the antimicrobial resistance patterns of human isolates with *S*. *aureus* isolates of animal origin, some differences were observed (Table 2). According to the CLSI (2012) [34] interpretation criteria, most *S*. *aureus* isolates from animals exhibited resistance to penicillin and tetracycline, and from retail meat to the former antibiotics and erythromycin. Tetracycline-resistant *S*. *aureus* strains were isolated from animals and retail meat, however, ciprofloxacin-resistant *S*. *aureus* strains were found in clinical isolates. A higher rate of MDR *S*. *aureus* strains were obtained from humans than animals and meat, which could be due to the high number of MRSA strains from clinical cases affected by acute infections that were included in this study. Most MRSA strains isolated from clinical cases have been found to be MDR [45]. In addition, clinical isolates (identified as MRSA) showed higher MICs to penicillin (Table 6) than *S*. *aureus* strains obtained from animals and meat [15] suggesting the potential influence of treatment or exposure on the selection of resistant strains. In this study, all *S*. *aureus* strains were susceptible to linezolid, which has been considered as a good alternative for the treatment of MDR *S*. *aureus* [46]. The CLSI (2012) [34] criteria establishes the susceptibility to daptomycin at MICs ≤1 μg/mL, therefore in clinical isolates MICs of 2 and 4 μg/mL were considered non-susceptible isolates. The interpretation of results for gentamicin, kanamycin, and penicillin could be ambiguous due to their accepted breakpoints and limited dilutions available on the NARMS panel. For example, all *S*. *aureus* strains isolated from humans exhibited MICs ≤128 μg/mL for gentamicin, which has a breakpoint ≥16 μg/mL; for kanamycin and penicillin some *S*. *aureus* strains showed MICs ≤128 μg/mL and ≤0.25 μg/mL, respectively, however the CLSI criteria recommends a breakpoint ≥64 μg/mL and ≥0.25 μg/mL as resistance. Therefore, interpretation of some resistances may require further evaluation using a wider dilution range of antimicrobials in order to improve the interpretation of susceptibility testing results for those antibiotics.

## Conclusion

The nasal carriage of *S*. *aureus* in healthy humans appears to be low, with clones genotypically diverse, and were *mecA*- and PVL-negative. *Staphylococcus aureus* strains harboring the *mecA* and PVL genes were present in clinical isolates from patients affected by invasive infections, and most of these isolates were of ST5 and ST8, and exhibited MDR profiles. Genetic similarity was observed between *S*. *aureus* strains isolated from humans and raw meat suggesting that contamination of meat during handling or processing could pose a risk for transmission to humans.

## Supporting Information

S1 Data MLST alleles and sequence types of selected human strains used in this study. (PDF)

S2 Data PCR data of healthy human isolates used in this study (PDF)

S3 Data PCR data of MRSA isolated from clinical isolates used in this study. (PDF)

S4 Data Antimicrobial susceptibility data for clinical and healthy human isolates examined in this study. (PDF)
