# Supplementary material for: Characterization of Staphylococcus aureus from Humans and a Comparison with İsolates of Animal Origin, in North Dakota, United States
Source: PLoS One. 2015 Oct 20;10(10):e0140497. doi: 10.1371/journal.pone.0140497 (PMC4618867; doi:10.1371/journal.pone.0140497)
Supplement: S1 Data — (PDF) [file pone.0140497.s001.pdf]

arcC		aroE		glpF	
A01	3	A03	4	A05	1
A02	3	A04	2	A06	1
B01	1	B03	1	B05	1
B02	2	B04	4	B06	2
C01	1	C03	3	C05	1
C02	1	C04	2	C06	8
D01	3	D03	4	D05	1
D02	8	D04	14	D06	1
E01	1	E03	4	E05	1
E02	10	E04	13	E06	2
F01	1	F03	1	F05	1
F02	13	F04	2	G04	1
G01	1	G03	4	G05	1
G02	2	H02	3	H04	1
H01	1	H03	3	H05	2

arcC	aroE	glpF	gmk	pta	tpi	yqiL
3	3	1	1	4	4	3
1	4	1	4	12	1	10
1	1	1	1	1	1	1
3	3	1	1	4	4	3
1	4	1	4	12	1	10
1	4	1	4	12	1	10
1	1	1	1	1	1	1
1	4	1	4	12	1	10
3	3	1	1	4	4	3
2	2	2	2	6	3	2
1	4	1	4	12	1	10
8	2	2	2	6	3	2
10	14	8	6	10	3	2
13	13	1	1	12	11	13
2	2	2	2	2	2	2

gmk		pta		tpi	
A07	1	A09	12	A11	1
A08	2	A10	10	A12	11
B07	4	B09	12	B11	1
B08	6	B10	12	B12	2
C07	4	C09	1	C11	1
C08	1	C10	2	D10	4
D07	1	D09	12	D11	4
D08	2	E08	4	E10	1
E07	4	E09	4	E11	3
F06	1	F08	12	F10	1
F07	1	F09	6	F11	1
G06	4	G08	1	G10	4
G07	2	G09	12	G11	3
H06	1	H08	4	H10	1
H07	4	H09	6	H11	3

ST	code
8	157
5	181
1	186
8	192
5	160
5	220
1	230
5	244
8	247
30	257
5	266
34	268
45	275
15	288
39	294

	yqiL
1	3
2	10
3	1
4	3
5	10
6	10
7	1
8	10
9	3
10	2
11	10
12	2
13	2
14	13
15	2
