# Supplementary material for: Characterization of Staphylococcus aureus from Humans and a Comparison with İsolates of Animal Origin, in North Dakota, United States
Source: PLoS One. 2015 Oct 20;10(10):e0140497. doi: 10.1371/journal.pone.0140497 (PMC4618867; doi:10.1371/journal.pone.0140497)
Supplement: S2 Data — (PDF) [file pone.0140497.s002.pdf]

Sample	Infection	Date	Staph	16S	mecA	Luk
1	Nasal	Fall 2010	epidermidis			
2	Nasal	Fall 2010	aureus	1	0	0
3	Nasal	Fall 2010	aureus	1	0	0
4	Nasal	Fall 2010	epidermidis			
5	Nasal	Fall 2010	aureus	1	0	0
6	Nasal	Fall 2010	epidermidis			
7	Nasal	Fall 2010	aureus	1	0	0
8	Nasal	Fall 2010	epidermidis			
9	Nasal	Fall 2010	epidermidis			
10	Nasal	Fall 2010	aureus	1	0	0
11	Nasal	Fall 2010	aureus	1	0	0
12	Nasal	Fall 2010	epidermidis			
13	Nasal	Fall 2010	aureus	1	0	0
14	Nasal	Fall 2010	aureus	1	0	0
15	Nasal	Fall 2010	aureus	1	0	0
16	Nasal	Fall 2010	aureus	1	0	0
17	Nasal	Fall 2010	warneri			
18	Nasal	Fall 2010	epidermidis			
19	Nasal	Fall 2010	aureus	1	0	0
20	Nasal	Fall 2010	warneri			
21	Nasal	Fall 2010	epidermidis			
22	Nasal	Fall 2010	epidermidis			
23	Nasal	Fall 2010	aureus	1	0	0
24DG	Nasal	Fall 2010	aureus	1	0	0
25	Nasal	Fall 2010	aureus	1	0	0
26	Nasal	Fall 2010	aureus	1	0	0
27	Nasal	Fall 2010	aureus	1	0	0
28	Nasal	Fall 2010	epidermidis			
29	Nasal	Fall 2010	aureus	1	0	0
30	Nasal	Spring 2011	aureus	1	0	0
31	Nasal	Spring 2011	aureus	1	0	0
32	Nasal	Spring 2011	aureus	1	0	0
33	Nasal	Spring 2011	aureus	1	0	0
34	Nasal	Spring 2011	aureus	1	0	0
35	Nasal	Spring 2011	aureus	1	0	0
36	Nasal	Spring 2011	aureus	1	0	0
37	Nasal	Spring 2011	aureus	1	0	0
38	Nasal	Spring 2011	epidermidis			
39	Nasal	Spring 2011	aureus	1	0	0
40	Nasal	Spring 2011	epidermidis			
41	Nasal	Spring 2011	aureus	1	0	0
42	Nasal	Spring 2011	aureus	1	0	0
43	Nasal	Spring 2011	aureus	1	0	0
44	Nasal	Spring 2011	aureus	1	0	0
45	Nasal	Spring 2011	aureus	1	0	0
46	Nasal	Spring 2011	aureus	1	0	0

47	Nasal	Spring 2011	aureus	1	0	0
48	Nasal	Spring 2011	epidermidis			
49	Nasal	Spring 2011	aureus	1	0	0
50	Nasal	Spring 2011	aureus	1	0	0
51	Nasal	Spring 2011	aureus	1	0	0
52	Nasal	Spring 2011	epidermidis			
53	Nasal	Spring 2011	aureus	1	0	0
54	Nasal	Spring 2011	aureus	1	0	0
55	Nasal	Spring 2011	aureus	1	0	0
56	Nasal	Spring 2011	epidermidis			
57	Nasal	Spring 2011	aureus	1	0	0
58	Nasal	Spring 2011	aureus	1	0	0
59	Nasal	Spring 2011	aureus	1	0	0
60	Nasal	Spring 2011	epidermidis			

29

31
