# Supplementary material for: Characterization of Staphylococcus aureus from Humans and a Comparison with İsolates of Animal Origin, in North Dakota, United States
Source: PLoS One. 2015 Oct 20;10(10):e0140497. doi: 10.1371/journal.pone.0140497 (PMC4618867; doi:10.1371/journal.pone.0140497)
Supplement: S3 Data — (PDF) [file pone.0140497.s003.pdf]

Sample	Cod Hospital	Infection	Date	Staph	16S	mecA	Luk
1	61-B	blood	19-07-2010	aureus	1	1	1
2	89-W	wound	19-07-2010	aureus	1	1	0
3	136-W	wound	19-07-2010	aureus	1	1	0
4	147-B	blood	19-07-2010	aureus	1	1	0
5	168-B	blood	19-07-2010	aureus	1	1	0
6	169-B	blood	19-07-2010	aureus	1	1	0
7	44-B	blood	19-07-2010	aureus	1	1	0
8	25-B	blood	19-07-2010	aureus	1	1	0
9	16-B	blood	19-07-2010	aureus	1	1	1
10	23-B	blood	19-07-2010	aureus	1	1	0
11	65-B	blood	19-07-2010	aureus	1	1	0
12	24-B	blood	19-07-2010	aureus	1	1	1
13	167-W	wound	19-07-2010	aureus	1	1	0
14	21-B	blood	19-07-2010	aureus	1	1	0
15	15-B	blood	19-07-2010	aureus	1	1	0
16	73-B	blood	19-07-2010	aureus	1	1	1
17	19-B	blood	19-07-2010	aureus	1	1	0
18	102-B	blood	19-07-2010	aureus	1	1	0
19	17-W	wound	19-07-2010	aureus	1	1	0
20	146-B1	blood	19-07-2010	aureus	1	1	0
21	78-B	blood	19-07-2010	aureus	1	1	0
22	98-B	blood	19-07-2010	aureus	1	1	0
23	129-B	blood	19-07-2010	aureus	1	1	0
24	106-B	blood	19-07-2010	aureus	1	1	0
25	27-B	blood	19-07-2010	aureus	1	1	0
26	18-W	wound	19-07-2010	aureus	1	1	0
27	134-B	blood	19-07-2010	aureus	1	1	0
28	138-W	wound	19-07-2010	aureus	1	1	0
29	31-B	blood	19-07-2010	aureus	1	1	0
30	137-B	blood	19-07-2010	aureus	1	1	0
31	28-B	blood	19-07-2010	aureus	1	1	0
32	103-B	blood	19-07-2010	aureus	1	1	0
33	56-B	blood	19-07-2010	aureus	1	1	0
34	55-B	blood	19-07-2010	aureus	1	1	0
35	64-B	blood	19-07-2010	aureus	1	1	0
36	57-B	blood	19-07-2010	aureus	1	0	0
37	90-B	blood	19-07-2010	aureus	1	1	1
38	94-B	blood	19-07-2010	aureus	1	1	0
39	99-B	blood	19-07-2010	aureus	1	1	0
40	146-B	blood	19-07-2010	aureus	1	1	0
41	166-B	blood	19-07-2010	aureus	1	1	0
42	72-B	blood	19-07-2010	aureus	1	1	0
43	24-B	blood	19-07-2010	aureus	1	1	0
44	41-B	blood	19-07-2010	aureus	1	1	0
45	130-B	blood	19-07-2010	aureus	1	1	0
46	104-B	blood	19-07-2010	aureus	1	1	0

47	62-B	blood	19-07-2010	aureus	1	1	0
48	71-B	blood	19-07-2010	aureus	1	1	0
49	42-B	blood	19-07-2010	aureus	1	1	0
50	128-B1	blood	19-07-2010	aureus	1	1	0
51	91-B	blood	19-07-2010	aureus	1	1	0
52	148-B	blood	19-07-2010	aureus	1	1	1
53	43-B	blood	19-07-2010	aureus	1	1	0
54	119-B	blood	19-07-2010	aureus	1	1	0
55	3-B	blood	19-07-2010	aureus	1	1	0
56	30-B	blood	19-07-2010	aureus	1	1	0
57	170-B	blood	19-07-2010	aureus	1	1	0
58	11-B	blood	19-07-2010	aureus	1	1	0
59	6-B	blood	19-07-2010	aureus	1	1	0
60	133-W	wound	19-07-2010	aureus	1	1	0
61	15-B	blood	19-07-2010	aureus	1	1	0
62	8-B	blood	19-07-2010	aureus	1	1	0
63	97-B	blood	19-07-2010	aureus	1	1	0
64	132-W	wound	19-07-2010	aureus	1	1	0
65	74-B	blood	19-07-2010	aureus	1	1	0
66	63-B	blood	19-07-2010	aureus	1	1	0
67	139-B	blood	19-07-2010	aureus	1	1	0
68	7-B	blood	19-07-2010	aureus	1	1	0
69	131-W2	wound	19-07-2010	aureus	1	1	0
70	34-B	blood	19-07-2010	aureus	1	1	1
71	29-B	blood	19-07-2010	aureus	1	1	0
72	40-B	blood	19-07-2010	aureus	1	1	0
73	96-B	blood	19-07-2010	aureus	1	1	0
74	49-B	blood	19-07-2010	aureus	1	1	1
75	92-B1	blood	19-07-2010	aureus	1	0	0
76	107-B	blood	19-07-2010	aureus	1	1	0
77	38-B	blood	19-07-2010	aureus	1	1	0
78	39-B	blood	19-07-2010	aureus	1	1	0
79	2-B	blood	19-07-2010	aureus	1	1	0
80	115-B	blood	19-07-2010	aureus	1	1	0
81	75-B	blood	19-07-2010	aureus	1	1	0
82	52-B	blood	19-07-2010	aureus	1	1	0
83	9-B	blood	19-07-2010	aureus	1	1	0
84	12-B	blood	19-07-2010	aureus	1	1	0
85	118-B	blood	19-07-2010	aureus	1	1	1
86	4-B	blood	19-07-2010	aureus	1	1	0
87	20-B	blood	19-07-2010	aureus	1	1	0
88	144-B	blood	19-07-2010	aureus	1	1	0
89	17-B	blood	19-07-2010	aureus	1	1	1
90	13-B	blood	19-07-2010	aureus	1	1	1
91	31-B	blood	19-07-2010	aureus	1	1	0
92	5-B	blood	19-07-2010	aureus	1	1	0
93	100-B	blood	19-07-2010	aureus	1	1	0

94	101-B	blood	19-07-2010	aureus	1	1	0
95	1-B	blood	19-07-2010	aureus	1	1	0
96	135-B	blood	19-07-2010	aureus	1	1	0
97	58-B	blood	19-07-2010	aureus	1	1	0
98	105-B	blood	19-07-2010	aureus	1	1	0
99	40-B	blood	19-07-2010	aureus	1	1	0
100	15-B	blood	19-07-2010	aureus	1	1	0
101	50-B	blood	19-07-2010	aureus	1	1	0
102	48-B	blood	19-07-2010	aureus	1	0	0
103	127-B	blood	19-07-2010	aureus	1	1	0
104	124-B	blood	19-07-2010	aureus	1	1	0
105	59-B	blood	19-07-2010	aureus	1	1	0
106	22-B	blood	19-07-2010	aureus	1	1	0
107	46-B	blood	19-07-2010	aureus	1	1	0
108	93-B	blood	19-07-2010	aureus	1	1	0
	Total blood	99					
	Total wound	9					
