# Supplementary material for: Characterization of Staphylococcus aureus from Humans and a Comparison with İsolates of Animal Origin, in North Dakota, United States
Source: PLoS One. 2015 Oct 20;10(10):e0140497. doi: 10.1371/journal.pone.0140497 (PMC4618867; doi:10.1371/journal.pone.0140497)
Supplement: S4 Data — (PDF) [file pone.0140497.s004.pdf]

New Code	Obs	Chl				Cip				Dap				Ery				Gen				Kan		
		R	I	S	NI	R	I	S	NI	R	I	S	NI	R	I	S	NI	R	I	S	NI	R	I	S
146	Clinical MRSA			8		>4						1		>8							≤128	>1024		
147	Clinical MRSA			8		>4						0,5		>8							≤128			
148	Clinical MRSA			8		>4						0,5		>8							≤128			
149	Clinical MRSA			8		>4						0,5		>8							≤128			
150	Clinical MRSA			8		>4						0,5				0,5					≤128			
151	Clinical MRSA			8		>4						0,5		>8							≤128			
152	Clinical MRSA			8		>4						0,5				≤0.25					≤128			
153	Clinical MRSA			8		>4						≤0.25		>8							≤128	1024		
154	Clinical MRSA			4		>4						0,5		>8							≤128	1024		
155	Clinical MRSA			8		>4						0,5		>8							≤128			
156	Clinical MRSA			8				0,25				0,5		>8							≤128			
157	Clinical MRSA			8				0,5				0,5		>8							≤128	>1024		
158	Clinical MRSA			8		>4						0,5		>8							≤128	>1024		
159	Clinical MRSA			8		>4						0,5		>8							≤128			
160	Clinical MRSA			8		>4						0,5		>8							≤128			
161	Clinical MRSA			8		>4						0,5		>8							≤128	>1024		
162	Clinical MRSA			8		>4						1		>8							≤128	256		
163	Clinical MRSA			8		>4						≤0.25		>8							≤128			
164	Clinical MRSA			8		>4						0,5		>8							≤128			
165	Clinical MRSA			8		>4						0,5		>8							≤128			
166	Clinical MRSA			8		>4						0,5		>8							≤128			
167	Clinical MRSA			8		>4						0,5		>8							≤128			
168	Clinical MRSA			8		>4						0,5		>8							≤128			
169	Clinical MRSA			8		>4						0,5		>8							≤128	>1024		
170	Clinical MRSA			8		>4						≤0.25		>8							≤128			
171	Clinical MRSA			8		>4						0,5		>8							≤128	256		
172	Clinical MRSA			8		>4						0,5		>8							≤128			
173	Clinical MRSA			8		>4						0,5		>8							≤128	>1024		
174	Clinical MRSA			8		>4						0,5		>8							≤128			
175	Clinical MRSA			4		>4						0,5		>8							≤128			
176	Clinical MRSA			8		>4						0,5		>8							≤128			
177	Clinical MRSA			8				0,5				0,5				0,5					≤128			
178	Clinical MRSA			8		>4						0,5		>8							≤128			
179	Clinical MRSA			4		>4						0,5		>8							≤128			
180	Clinical MRSA			8		>4				2				>8							≤128	1024		
181	Clinical MRSA			8		>4				4				>8							≤128			
182	Clinical MRSA			8				0,5				0,5		>8							≤128	>1024		
183	Clinical MRSA		16			>4						0,5		>8							≤128			
184	Clinical MRSA			8		>4						≤0.25		>8							≤128			
185	Clinical MRSA			8		>4						0,5		>8							≤128			
186	Clinical MRSA			8		>4						0,5				0,5					≤128			
187	Clinical MRSA			4		>4						0,5			4						≤128	1024		
188	Clinical MRSA			8		>4						0,5		>8							≤128	512		
189	Clinical MRSA			8		>4						0,5		>8							≤128			
190	Clinical MRSA		16			>4						0,5		>8							≤128			
191	Clinical MRSA			8		>4						0,5		>8							≤128			
192	Clinical MRSA			8		>4						0,5				≤0.25					≤128			
193	Clinical MRSA			8		>4						1		>8							≤128			
194	Clinical MRSA			8		>4						0,5		>8							≤128			

195	Clinical MRSA			8		>4						0,5		>8							≤128			
196	Clinical MRSA			8		>4						0,5		>8							≤128			
197	Clinical MRSA			8				0,5				0,5				0,5					≤128			
198	Clinical MRSA			4		>4						≤0.25		>8							≤128			
199	Clinical MRSA			8		>4						0,5		>8							≤128			
200	Clinical MRSA			8		>4						1		>8							≤128			
201	Clinical MRSA			8				0,25				≤0.25				0,5					≤128			
202	Clinical MRSA			8		>4						0,5		>8							≤128			
203	Clinical MRSA			8				0,5				0,5				0,5					≤128			
204	Clinical MRSA			8		>4						0,5		>8							≤128			
205	Clinical MRSA			4		>4				2				8							≤128	1024		
206	Clinical MRSA			8		>4						0,5		>8							≤128			
207	Clinical MRSA			8		>4						0,5		>8							≤128	256		
208	Clinical MRSA			8		>4				4				>8							≤128	512		
209	Clinical MRSA			8		>4				2				>8							≤128	1024		
210	Clinical MRSA			8		>4				4				>8							≤128	512		
211	Clinical MRSA			4		>4						0,5		>8							≤128	256		
212	Clinical MRSA			8		>4						0,5		>8							≤128	>1024		
213	Clinical MRSA			8		>4						0,5		>8							≤128	1024		
214	Clinical MRSA			8		>4						0,5		>8							≤128			
215	Clinical MRSA			8		>4						0,5		>8							≤128	>1024		
216	Clinical MRSA			8		>4						0,5		>8							≤128	512		
217	Clinical MRSA			8		>4						0,5		>8							≤128	256		
218	Clinical MRSA			8		>4						0,5				0,5					≤128			
219	Clinical MRSA			8		>4						≤0.25		>8							≤128	1024		
220	Clinical MRSA			8		>4						1		>8							≤128	512		
221	Clinical MRSA			8		>4						0,5		>8							≤128			
222	Clinical MRSA			4		>4						≤0.25		>8							≤128			
223	Clinical MRSA			4		>4						≤0.25				0,5					≤128			
224	Clinical MRSA			8		>4						0,5		>8							≤128	1024		
225	Clinical MRSA			8		>4						0,5		>8							≤128	1024		
226	Clinical MRSA			8		>4						0,5		>8							≤128	1024		
227	Clinical MRSA			8		>4						0,5		>8							≤128	512		
228	Clinical MRSA			8		>4						0,5		>8							≤128	512		
229	Clinical MRSA			8		>4						≤0.25		>8							≤128			
230	Clinical MRSA			8				0,25				0,5			1						≤128			
231	Clinical MRSA			8				0,25				1		>8							≤128			
232	Clinical MRSA			8		>4						0,5		>8							≤128			
233	Clinical MRSA			8		>4						0,5		>8							≤128			
234	Clinical MRSA			8		>4						0,5		>8							≤128	256		
235	Clinical MRSA			8		>4						0,5		>8							≤128	1024		
236	Clinical MRSA			8		>4						0,5		>8							≤128			
237	Clinical MRSA			8		>4						0,5		>8							≤128			
238	Clinical MRSA			8				1				0,5		8							≤128			
239	Clinical MRSA			8		>4						0,5		>8							≤128			
240	Clinical MRSA			8		>4						0,5		>8							≤128			
241	Clinical MRSA			8		>4						0,5		>8							≤128			
242	Clinical MRSA			8		>4						1		>8							≤128	256		
243	Clinical MRSA			8		>4						≤0.25		>8							≤128	1024		
244	Clinical MRSA			8				0,5				≤0.25				0,5					≤128			
245	Clinical MRSA			8		>4						0,5		>8							≤128			

246	Clinical MRSA			8		>4						0,5		>8							≤128	256		
247	Clinical MRSA			8		>4						0,5		8							≤128	256		
248	Clinical MRSA			4				1				0,5			4						≤128			
249	Clinical MRSA			8		>4						≤0.25		>8							≤128	256		
250	Clinical MRSA			8		>4						0,5		>8							≤128			
251	Clinical MRSA			8		>4						≤0.25		>8							≤128			
252	Clinical MRSA			8		>4						0,5		8							≤128			
253	Clinical MRSA			8		>4						≤0.25		>8							≤128			
254	Healthy			8				0,25				0,5				0,5					≤128			
255	Healthy			4		>4						0,5		>8							≤128			
256	Healthy			8				0,25				≤0.25				0,5					≤128			
257	Healthy			8		>4						≤0.25			2						≤128	256		
258	Healthy			8				0,5				≤0.25			1						≤128			
259	Healthy			8		>4						≤0.25			4						≤128			
260	Healthy			8			2					≤0.25		>8							≤128			
261	Healthy			8				0,25				≤0.25			1						≤128			
262	Healthy			8				0,25				≤0.25		>8							≤128			
263	Healthy			8				0,5				0,5				0,5					≤128			
264	Healthy			8				0,5				0,5		>8							≤128			
265	Healthy			8				0,25				≤0.25		>8							≤128			
266	Healthy			8		>4						0,5		>8							≤128	256		
267	Healthy			8				1				0,5			2						≤128			
268	Healthy			8		>4						0,5		>8							≤128	512		
269	Healthy			8		>4						1		>8							≤128	512		
270	Healthy			8				0,25				0,5		>8							≤128			
271	Healthy			8				0,25				0,5				0,5					≤128			
272	Healthy			8			2					≤0.25				≤0.25					≤128			
273	Healthy			8		>4						0,5				0,5					≤128			
274	Healthy			4				0,25				≤0.25				0,5					≤128			
275	Healthy			8		>4						≤0.25			2						≤128			
276	Healthy			8		>4						≤0.25		8							≤128			
277	Healthy			4				0,25				1				0,5					≤128			
278	Healthy			8		>4						0,5		>8							≤128	512		
279	Healthy			4				0,5				≤0.25		>8							≤128			
280	Healthy			8				0,5				≤0.25				≤0.25					≤128			
281	Healthy			8				0,5				≤0.25				≤0.25					≤128			
282	Healthy			8				0,5				0,5				≤0.25					≤128			
283	Healthy			4				≤0.25				≤0.25		>8							≤128			
284	Healthy			8		>4						≤0.25			4						≤128			
285	Healthy			8				≤0.12				≤0.25				0,5					≤128			
286	Healthy			8				0,5				≤0.25				0,5					≤128			
287	Healthy			8		>4						0,5		>8							≤128			
288	Healthy			8				0,5				0,5		8							≤128	256		
289	Healthy			8				0,25				0,5		>8							≤128			
290	Healthy		16			4						≤0.25		>8							≤128			
291	Healthy			4		>4						≤0.25		>8							≤128	1024		
292	Healthy			8				0,5				0,5		>8							≤128			
293	Healthy			8		>4						0,5			4						≤128			
294	Healthy			8				0,5				0,5		8							≤128			
295	Healthy			8				1				0,5				0,5					≤128			

	Linc				Line				Nit				Pen				Qui				Str				Tet				
NI	R	I	S	NI	R	I	S	NI	R	I	S	NI	R	I	S	NI	R	I	S	NI	R	I	S	NI	R	I	S	NI	R
				≤1			2				16		>16						≤0.5					≤512			≤1		
≤128				≤1			2				16		16						≤0.5					≤512			≤1		
≤128				>8			2				16		8						≤0.5					≤512			≤1		
≤128				>8			2				16		16						≤0.5					≤512			≤1		
≤128				≤1			2				16		16						≤0.5					≤512			≤1		
≤128				>8			2				16		8						≤0.5					≤512			≤1		
≤128				≤1			2				8					≤0.25			≤0.5					≤512			≤1		
				≤1			2				8		>16						≤0.5					≤512			≤1		
				≤1			1				16		16						≤0.5					≤512			≤1		
≤128				>8			1				16		>16						≤0.5					≤512			≤1		
≤128				≤1			2				16		16						≤0.5					≤512			≤1		
				≤1			2				16		>16						≤0.5					≤512			≤1		
				≤1			2				16		8						≤0.5					≤512			≤1		
≤128				>8			2				8		4						≤0.5					≤512			≤1		
≤128				≤1			2				8		8						≤0.5					≤512			≤1		
				≤1			2				16		>16						≤0.5					≤512			≤1		
				>8			2				16		1						≤0.5					≤512			≤1		
≤128				>8			2				16		16						≤0.5					≤512			≤1		
≤128				>8			2				16		4						≤0.5					≤512			≤1		
≤128				>8			1				16		4						≤0.5					≤512			≤1		
≤128				>8			2				16		4						≤0.5					≤512			≤1		
≤128				>8			2				16		8						≤0.5					≤512			≤1		
≤128				>8			2				16		4						≤0.5					≤512			≤1		
				>8			2				16		>16						≤0.5					≤512	16				
≤128				>8			1				16		8						≤0.5					≤512			≤1		
				>8			2				16		4						≤0.5					≤512			≤1		
≤128				2			2				16		8						≤0.5					≤512			≤1		
				≤1			2				16		>16						≤0.5					≤512			≤1		
≤128				≤1			2				16		8						≤0.5					≤512			≤1		
≤128				≤1			1				8		8						≤0.5					≤512			≤1		
≤128				≤1			2				16		2						≤0.5					≤512			≤1		
≤128				≤1			2				16		>16						≤0.5					≤512			≤1		
≤128				>8			1				8		4						≤0.5					≤512			≤1		
≤128				≤1			1				16		4						≤0.5					≤512			≤1		
				>8			2				16		16						≤0.5					≤512			≤1		
≤128				>8			2				16		8						≤0.5					≤512			≤1		
				≤1			2				16		16						≤0.5					≤512			≤1		
≤128				>8			2				16		8						≤0.5					≤512			≤1		
≤128				>8			1				16		8						≤0.5					≤512			≤1		
≤128				>8			1				16		8						≤0.5					≤512			≤1		
≤128				≤1			1				4					≤0.25			≤0.5					≤512			≤1		
				≤1			1				8		8						≤0.5					≤512			≤1		
				≤1			2				8					≤0.25			≤0.5					≤512			≤1		
≤128				>8			1				4		1						≤0.5					≤512			≤1		
≤128				>8			2				8		8						≤0.5					≤512			≤1		
≤128				>8			1				8		4						≤0.5					≤512			≤1		
≤128				≤1			2				16		>16						≤0.5					≤512	32				
≤128				≤1			2				8					≤0.25			≤0.5					≤512			≤1		
≤128				>8			1				8		2						≤0.5					≤512			≤1		

≤128				≤1			1				16		8						≤0.5					≤512			≤1		
≤128				>8			1				8		4						≤0.5					≤512			≤1		
≤128				≤1			2				16		16						≤0.5					≤512			≤1		
≤128				>8			≤0.5				4		8						≤0.5					≤512			≤1		
≤128				>8			2				32		4						≤0.5					≤512			≤1		
≤128				≤1			2				8		1						≤0.5					≤512			≤1		
≤128				≤1			2				8					≤0.25			≤0.5					≤512			≤1		
≤128				>8			2				8		16						≤0.5					≤512			≤1		
≤128				≤1			2				8		8						≤0.5					≤512			≤1		
≤128				>8			2				16		8						≤0.5					≤512			≤1		
				≤1			≤0.5				8		16						≤0.5					≤512			≤1		
≤128				>8			2				16		16						≤0.5					≤512			≤1		
				≤1			2				16		16						≤0.5					≤512			≤1		
				>8			2				16		1						≤0.5					≤512			≤1		
				>8			2				16		16						≤0.5					≤512			≤1		
				>8			1				8		4						≤0.5					≤512			≤1		
				8			1				8		>16						≤0.5					≤512			≤1		
				>8			2				16		>16						≤0.5					≤512			≤1		
				>8			2				16		4						≤0.5					≤512			≤1		
≤128				2			1				16		8						≤0.5					≤512			≤1		
				≤1			2				16		>16						≤0.5					≤512			≤1		
				>8			1				16		>16						≤0.5					≤512	32				
				>8			2				16		16				>16							≤512		8			
≤128				≤1			2				8		>16						≤0.5					≤512			≤1		
				≤1			2				16		>16						≤0.5					≤512			≤1		
				>8			2				16		8						≤0.5					≤512			≤1		
≤128				>8			2				16		8						≤0.5					≤512			≤1		
≤128				>8			2				16		>16						≤0.5					≤512			≤1		
≤128				≤1			1				16		>16						≤0.5					≤512			≤1		
				>8			2				16		8						≤0.5					≤512			≤1		
				>8			2				16		16						≤0.5					≤512			≤1		
				>8			2				16		16						≤0.5					≤512			≤1		
				>8			1				16		4						≤0.5					≤512			≤1		
				≤1			2				16		16						≤0.5					≤512			≤1		
≤128				>8			2				16		16						≤0.5					≤512			≤1		
≤128				≤1			2				8		16						≤0.5					≤512			≤1		
≤128				≤1			2				16		16						≤0.5					≤512			≤1		
≤128				≤1			2				16		16						≤0.5					≤512			≤1		
≤128				>8			2				16		>16						≤0.5					≤512			≤1		
				>8			2				16		16						≤0.5					≤512			≤1		
				2			2				16		>16						≤0.5					≤512			≤1		
≤128				>8			2				16		8						≤0.5					≤512			≤1		
≤128				≤1			2				16		16						≤0.5					≤512			≤1		
≤128				≤1			2				16		2						≤0.5					≤512			≤1		
≤128				>8			1				16		16						≤0.5					≤512			≤1		
≤128				>8			2				16		16						≤0.5					≤512			≤1		
≤128				>8			1				16		>16						≤0.5					≤512			≤1		
				>8			2				16		16						≤0.5					≤512			≤1		
				≤1			2				16		16						≤0.5					≤512			≤1		
≤128				≤1			2				16		4						≤0.5					≤512			≤1		
≤128				≤1			2				16		8						≤0.5					≤512			≤1		

				≤1			2				16		>16						≤0.5					≤512			≤1		
				>8			2				8		>16						≤0.5					≤512			≤1		
≤128				≤1			1				8		>16						≤0.5					≤512			≤1		
				>8			1				16		8						≤0.5					≤512			≤1		
≤128				≤1			2				8		16						≤0.5					≤512			≤1		
≤128				>8			2				16		16						≤0.5					≤512			≤1		
≤128				>8			2				16		>16						≤0.5					≤512			≤1		
≤128				>8			2				16		>16						≤0.5					≤512			≤1		
≤128				≤1			2				8		2						≤0.5					≤512			≤1		
≤128				>8			1				8		4						≤0.5					≤512			≤1		
≤128				≤1			2				16		4						≤0.5					≤512			≤1		
				4			2				16		8						≤0.5					≤512			≤1		
≤128				≤1			2				16		0,5						≤0.5					≤512			≤1		
≤128				≤1			2				8		4						≤0.5					≤512			≤1		
≤128				≤1			2				8		4						≤0.5					≤512			≤1		
≤128				≤1			2				16		0,5						≤0.5					≤512			≤1		
≤128				2			2				16					≤0.25			≤0.5					≤512			4		
≤128				≤1			2				16		0,5						≤0.5					≤512			≤1		
≤128				8			2				8		16						≤0.5					≤512	16				
≤128				>8			1				16		4						≤0.5					≤512	>32				
				>8			2				16		2						≤0.5					≤512			≤1		
≤128				≤1			2				16		2						≤0.5					≤512			≤1		
				>8			2				16		16						≤0.5					≤512			≤1		
				>8			1				8		>16						≤0.5					≤512	>32				
≤128				≤1			2				16		4						≤0.5					≤512			≤1		
≤128				≤1			2				16		4						≤0.5					≤512			≤1		
≤128				≤1			2				16		4						≤0.5					≤512			≤1		
≤128				≤1			2				8		8						≤0.5					≤512			≤1		
≤128				≤1			2				8		2						≤0.5					≤512			≤1		
≤128				≤1			2				8		2						≤0.5					≤512			≤1		
≤128				≤1			2				16		8						≤0.5					≤512			≤1		
≤128				≤1			≤0.5				8		0,5						≤0.5					≤512			≤1		
				8			2				16		8						≤0.5					≤512	>32				
≤128				2			2				8					≤0.25			≤0.5					≤512			2		
≤128				≤1			2				8		8						≤0.5					≤512			≤1		
≤128				≤1			2				16		0,5						≤0.5					≤512			≤1		
≤128				≤1			2				16		8						≤0.5					≤512			≤1		
≤128				≤1			1				8		2						≤0.5					≤512			≤1		
≤128				≤1			2				16		4						≤0.5					≤512			4		
≤128				≤1			2				16		4						≤0.5					≤512			≤1		
≤128				≤1			2				16		0,5						≤0.5					≤512			≤1		
≤128				≤1			2				16		8						≤0.5					≤512			≤1		
				2			2				16		16						1					≤512			≤1		
≤128				≤1			2				16		8						≤0.5					≤512			≤1		
≤128				>8			2				16		16						≤0.5					≤512			≤1		
				4			1				16		8						≤0.5					≤512	>32				
≤128				≤1			2				16		4						≤0.5					≤512			≤1		
≤128				≤1			2				16		8						≤0.5					≤512			≤1		
≤128				2			2				16		0,5						≤0.5					≤512			≤1		
≤128				≤1			2				16		16						≤0.5					≤512			≤1		

Tig			Tyl				Van				
I	S	NI	R	I	S	NI	R	I	S	NI	Total Resist
		0,25				2			1		4
		0,5				2			1		3
		0,5				>32			1		3
		0,5				>32			1		3
		0,5				1			1		2
		0,06				>32			1		3
		0,06				1			1		1
		0,06				1			1		4
		0,06				2			1		4
		0,12				>32			1		3
		>0.5				1			1		2
		0,25				2			1		3
		0,12				2			1		4
		0,12				>32			1		3
		0,25				1			1		3
		0,5				2			1		4
		0,25				>32			2		4
		0,12				>32			1		3
		0,25				>32			2		3
		0,12				>32			1		3
		0,25				>32			1		3
		0,5				>32			1		3
		0,25				>32			1		3
		0,25				>23			1		5
		0,12				>32			1		3
		0,25				>32			2		4
		0,25				4			1		3
		0,25				2			2		4
		0,12				1			1		3
		0,06				1			1		3
		0,12				1			1		3
		0,25				2			1		1
		0,5				>32			1		3
		0,12				1			1		3
		0,12				>32			2		5
		0,5				>32			2		4
		0,25				1			1		3
		0,25				>32			1		3
		0,25				>32			1		3
		0,25				>32			1		3
		0,25				1			1		1
		0,5				1			1		3
		0,12				1			1		3
		0,5				>32			1		3
		0,12				>32			1		3
		0,25				>32			1		3
		0,12				1			1		3
		0,12				1			2		2
		0,12				>32			2		3

		0,5				1			1		3
		0,5				>32			1		3
		0,5				1			1		1
		0,12				>32			0,5		3
		0,5				>32			1		3
		0,25				0,5			2		3
		0,5				1			1		0
		0,5				>32			2		3
		0,5				1			1		1
		0,5				>32			1		3
		0,25				1			2		5
		0,5				>32			1		3
		0,5				1			1		4
		0,12				32			2		5
		0,25				>32			2		5
		0,5				>32			0,5		5
		0,5				1			2		4
		0,25				>32			1		4
		0,5				>32			1		4
		0,12				1			1		3
		0,5				2			1		4
		0,5				>32			1		5
		0,5				>32			1		5
		0,25				1			1		2
		0,12				2			1		4
		0,06				32			2		4
		0,25				32			1		3
		0,12				>32			1		3
		0,12				2			1		2
		0,5				>32			1		4
		0,12				>32			1		4
		0,25				>32			1		4
		0,12				>32			1		4
		0,12				1			1		4
		0,25				>32			1		3
		0,5				2			1		1
		0,5				2			1		2
		0,5				2			1		3
		0,5				>32			1		3
		0,5				>32			2		4
		0,12				2			1		4
		0,5				>32			1		3
		0,12				1			1		3
		0,25				1			1		2
		0,25				>32			1		3
		0,25				>32			1		3
		0,5				>32			1		3
		0,12				>32			1		4
		0,06				1			1		4
		0,06				2			1		1
		0,25				1			1		3

		0,5				32			2		4
		0,5				8			1		4
		0,12				2			1		1
		0,12				>32			1		4
		0,12				>32			1		3
		0,25				>32			1		3
		0,12				2			1		3
		0,25				1			1		3
		>.5				2			1		1
		0,5				>32			1		3
		0,12				1			1		1
		0,12				16			1		3
		0,12				4			1		1
		0,25				1			1		2
		0,12				2			1		2
		0,12				2			1		1
		0,12				4			1		1
		0,12				1			1		1
		0,25				4			1		3
		0,5				>32			1		3
		0,12				>32			1		4
		0,25				16			1		1
		0,25				>32			1		4
		0,25				>32			2		5
		0,5				2			1		2
		0,25				2			1		1
		0,12				1			1		1
		0,12				2			1		2
		0,12				1			1		1
		0,12				1			1		2
		0,12				4			1		3
		0,25				4			1		1
		0,25				8			1		5
		0,12				1			1		1
		0,25				2			1		1
		0,06				1			1		1
		0,25				2			1		1
		0,25				2			1		2
		0,12				8			1		2
		0,12				1			1		1
		0,25				2			1		1
		0,5				2			1		3
		0,06				8			1		3
		0,06				2			1		2
		0,25				>32			1		3
		0,12				1			1		5
		0,25				2			1		2
		0,25				2			1		2
		0,12				1			1		2
		0,12				2			1		1

1 TOTAL 0

											26	TOTAL 1
											18	TOTAL 2
											64	TOTAL 3
											30	TOTAL 4
											11	TOTAL 5
											0	TOTAL 6
											0	TOTAL 7
											0	TOTAL 8
											150	
